# The Complete Genomes of *Microcystis ichthyoblabe* Kützing and *Microcystis protocystis* (Crow) Komárek & Anagnostidis Reveal the Complexity and Plasticity of *Microcystis* Genomes

**DOI:** 10.3390/microorganisms13071693

**Published:** 2025-07-18

**Authors:** Jina Kim, Hyaekang Kim, Jaeduk Goh, Seung Won Nam, Eu Jin Chung, Miyoung Shin, Donghyeok Seol, Ki Hwan Kim, Woori Kwak

**Affiliations:** 1 Department of Biotechnology, The Catholic University of Korea, Bucheon 14662, Republic of Korea; 2 Bio-Resources Bank Division, Nakdonggang National Institute of Biological Resources, Sangju 37242, Republic of Korea; 3 Prokaryote Research Division, Biological Resources Research Department, Nakdonggang National Institute of Biological Resources, Sangju 37242, Republic of Korea; 4 Department of Pathology, Yale University School of Medicine, New Haven, CT 06510, USA; 5 Department of Surgery, Seoul National University Bundang Hospital, Bundang-Gu, Seongnam 13620, Republic of Korea; 6 Gencube Plus, Seoul 08592, Republic of Korea; 7 Department of Medical and Biological Sciences, The Catholic University of Korea, Bucheon 14662, Republic of Korea

**Keywords:** *Microcystis*, morphospecies, complete genome

## Abstract

*Microcystis* is a genus of cyanobacteria responsible for harmful algal blooms (HABs) in freshwater ecosystems, posing significant ecological and public health risks. Despite its importance, current genomic resources are heavily biased toward *Microcystis aeruginosa*, limiting comprehensive understanding of genomic diversity within the genus. In this study, we present the first complete genome sequences of two morphospecies, *M. ichthyoblabe* FBCC-A1114 and *M. protocystis* FBCC-A270. Using long-read sequencing, both genomes were assembled into single circular chromosomes of 5.84 Mb and 5.76 Mb, respectively. Phylogenetic analyses placed both strains within genospecies G, alongside *M. aeruginosa* and *M. viridis*. Comparative analysis of biosynthetic gene clusters revealed that, while most genospecies G members harbor aeruginosin, cyanobactin, and microviridin gene clusters, the two newly sequenced strains lack cyanobactin and microcystin clusters but retain the microginin cluster. Synteny analysis demonstrated high structural conservation between the two genomes, while notable structural variations were observed when compared with *M. aeruginosa* NIES-298. These findings reveal both functional and structural plasticity within the genospecies, suggesting ecotype diversification driven by environmental adaptation. The newly assembled genomes provide critical resources to refine classification frameworks and advance our understanding of *Microcystis* genomic diversity.

## 1. Introduction

*Microcystis* is a genus of cyanobacteria that proliferates as phytoplankton in eutrophic freshwater environments such as lakes, reservoirs, and rivers [1,2]. Under nitrogen- and phosphorus-rich conditions, its rapid growth triggers harmful algal blooms (HABs), forming dense green mats on the water surface and significantly impairing aquatic ecosystem health [3]. *Microcystis* produces microcystin, a cyclic heptapeptide, with over 300 structural variants reported to date; among them, microcystin-LR is the most prevalent and toxic [4,5]. Microcystins inhibit protein phosphatases, leading to hepatocellular damage, acute liver injury, and failure upon high-dose exposure. Chronic exposure to microcystins is associated with an increased risk of hepatocellular carcinoma and has also been linked to toxicity in the kidneys, cardiovascular system, and nervous system [6,7,8,9,10]. Environmentally, microcystins contaminate freshwater ecosystems, causing mass mortality of fish and aquatic invertebrates [11]. Recent findings have shown that microcystins can be transported into marine environments and cause mortality in predators such as sea otters, suggesting that the ecological impacts of HABs may extend beyond freshwater systems [12]. Consequently, contamination of drinking water sources poses significant public health risks and elevates water treatment costs [13,14,15]. Given these escalating concerns, there is a growing interest in establishing a robust classification framework for management of *Microcystis*.

Traditionally, classification of *Microcystis* has been described based on colony morphology, cell size, and mucilage structure [16,17,18,19]. However, changes in morphospecies during cultivation have been reported [20,21], and morphological traits can vary in response to environmental factors such as temperature, nutrient concentration, and light availability [22,23,24]. Due to this plasticity, morphology-based classification is insufficient for accurate species delineation within *Microcystis*. To overcome these limitations, molecular markers including 16S rDNA have been introduced, but high sequence similarity among morphospecies continues to pose challenges [1,25,26]. For instance, the highly variable internal transcribed spacer (ITS) region shows 93.9–100% sequence identity across 47 *Microcystis* strains, with morphospecies often clustering inconsistently or being dispersed across multiple clades within phylogenetic trees [27]. Such incongruences between morphological and molecular classifications underscore the need for comprehensive framework.

Recent studies have adopted approaches that combine phylogenetic and other methods to more accurately define species boundaries [28]. One of these studies classified 122 *Microcystis* strains into 16 genospecies by applying pan-genome analysis in conjunction with orthologous average genome relatedness index (OGRI) thresholds, which were determined based on average nucleotide identity (ANI) and digital DNA–DNA hybridization (dDDH) values [28,29]. This framework, incorporating both core and non-core gene content, reflects the evolutionary dynamics of *Microcystis* such as vertical inheritance and horizontal gene transfer. Despite these advances, taxonomic inconsistencies persist due to limited genomic data for several morphospecies.

The persistent incongruence between morphology-based and genome-based classifications has posed a substantial taxonomic challenge in the genus *Microcystis*. Morphospecies that are readily distinguishable by colony structure or mucilage traits are often indistinguishable at the genomic level, and conversely, genomically divergent strains may exhibit highly similar morphological features. This mismatch complicates species delineation, ecological monitoring, and toxicity prediction, as accurate identification of *Microcystis* strains is critical for managing HABs. Among these morphospecies, *M. ichthyoblabe* and *M. protocystis* have remained particularly underrepresented in genomic studies, leaving a key knowledge gap in understanding their evolutionary placement and genomic traits. The absence of complete genome sequences for these morphospecies has hindered efforts to determine whether their distinct morphological identities correspond to unique genospecies or are manifestations of phenotypic plasticity within a broader genomic lineage.

In this study, we report the first complete genome sequences of *M. ichthyoblabe* and *M. protocystis*, which had not been previously reported. Through comparative genome analyses, we aimed to expand our understanding of the evolutionary dynamics of *Microcystis*. Based on the comprehensive framework proposed by Cai et al., (2023), we evaluated the genospecies classification of the assembled strains using phylogenetic analysis and OGRI thresholds [29]. Furthermore, we predicted biosynthetic gene clusters and compared genome structures with strains of the same genospecies, thereby assessing their genetic and functional complexity. The addition of genomic information not only enriches the current dataset but also provides a broader perspective on genetic plasticity within *Microcystis*. These findings are expected to help resolve incongruences between morphological and genomic classification.

## 2. Materials and Methods

### 2.1. Strain Collection and Culture Conditions

*M. aeruginosa* FBCC-A68, *M. ichthyoblabe* FBCC-A1114, and *M. protocystis* FBCC-A270 were obtained from the Freshwater Bioresources Culture Collection (FBCC), Republic of Korea. The original environmental samples were collected from freshwater ponds: FBCC-A68 from Gashiyeon Wetland (37°47′20.6″ N, 128°53′51.7″ E) in June 2018, FBCC-A1114 and FBCC-A270 from Geum River (36°21′10.0″ N, 127°33′45.9″ E and 36°20′28.3″ N, 127°33′32.2″ E) in July 2018. Each strain was isolated into unialgal culture and grown in Blue-Green Medium (BG11) at 20 °C under a 16:8 h light:dark cycle with a light intensity of 3000 lux provided by cool-white fluorescent lamps.

### 2.2. DNA Extraction and Library Preparation

High-quality genomic DNA was extracted using the Mag-Bind^®^ Universal Pathogen Kit (Omega Bio-Tek, Norcross, GA, USA) according to the manufacturer’s protocol. Genomic DNA purity and concentration were measured using a NanoDrop 2000 Spectrophotometer (Thermo Fisher Scientific, Waltham, MA, USA) and a Qubit 4 Fluorometer (Thermo Fisher Scientific, Waltham, MA, USA) with the dsDNA High Sensitivity Assay Kit. Long-read libraries were prepared with the Oxford Nanopore Technologies (ONT, Oxford, UK) ligation sequencing kit (SQK-LSK114, ONT) and sequenced on a Flongle R10.4 flow cell (FLO-FLG114, ONT). Short-read libraries were prepared using the Illumina TruSeq Nano DNA kit and sequenced on the Illumina NovaSeq 6000 platform (Illumina, San Diego, CA, USA), generating paired-end 150 bp reads for hybrid genome polishing.

### 2.3. Genome Sequencing and Assembly

Generated Nanopore reads from Flongle were basecalled and adapter-trimmed using Dorado v0.8.0 (https://github.com/nanoporetech/dorado, accessed on 15 July 2024) with the --trim adapters parameter and super accuracy model V5, and genome assembly was performed using Flye v2.9.5 [30] with --nano-hq parameter. In the case of *Microcystis aeruginosa* A68, the presence of symbiotic microorganisms was detected in the initial genome assembly. Therefore, the genome of *M. aeruginosa* A68 was obtained using a meta-assembly approach with the --meta parameter in Flye. All assembled genomes underwent two-step polishing: first, Medaka v2.0.1 (https://github.com/nanoporetech/medaka, accessed on 15 June 2025) was used to improve consensus accuracy with Nanopore reads; second, Pilon v1.24 [31] further refined the assemblies using Illumina reads with default parameter. Short reads were mapped to assembled genome using Bowtie2 [32] with --no-mixed parameter. Genome completeness was assessed using BUSCO v5.8.2 [33] with the chroococcales_odb10 dataset, and structural integrity and contamination were evaluated with CheckM2 v1.1.0 [34]. Species identification was validated using JSpeciesWS [35] via Tetra Correlation Search (TCS). Gene prediction and annotation were conducted with Prokka v1.14.5 [36]. Generated data and assembled genomes were deposited to NCBI database under project accession PRJNA1262101. The number of raw bases before and after adapter trimming for each genome is provided in Supplementary Table S1.

### 2.4. Phylogenetic Analysis

Phylogenetic tree construction and genospecies thresholding based on OGRI were conducted following the framework proposed by Cai et al., (2023) [28,29]. Pan-genome analysis was carried out using PEPPAN v1.0.5 [37], with GFF files from Prokka as input. The PEPPAN_parser module provided both the gene presence/absence matrix and single-copy core gene sequences. These sequences were aligned using MAFFT v7.526 [38], concatenated with FASconCAT-G v1.06.1 [39], and used to infer a maximum likelihood phylogeny in RAxML-NG v1.2.2 [40] (GTR + G model, 1000 bootstraps). OGRI metrics were calculated using pyani v0.2.10 [41] to determine ANI and the GGDC web server to estimate dDDH values [42]. A comprehensive list of all *Microcystis* genomes used in analysis, including their accession numbers, genome features, geographic origins, and assigned genospecies, is provided in Supplementary Table S2.

### 2.5. Secondary Metabolite Biosynthetic Gene Cluster and Genome Structure Analysis

Secondary metabolite biosynthetic gene clusters (BGCs) were predicted using antiSMASH v7.1.0 [43] with strict mode enabled, and results were visualized via custom R scripts [44] using the ggplot2 package v3.5.2 [45]. Genome structural comparisons were conducted using Mauve v2.4.0 [46], employing the progressive alignment mode for synteny analysis.

## 3. Results and Discussion

### 3.1. Complete Genome Assembly of Three Microcystis Strains

Obtaining high-quality, complete genomes from cyanobacteria is challenging due to symbiotic contaminants in non-axenic cultures [47,48]. To address this, we isolated three *Microcystis* strains (*M. aeruginosa* FBCC-A68, *M. ichthyoblabe* FBCC-A1114, and *M. protocystis* FBCC-A270) to establish unialgal cultures and performed sequencing their genomes using a hybrid long-read and short-read approach. Hybrid assembly resolved structural ambiguities and minimized contamination from coexisting microorganisms, while a two-step polishing strategy—initial error correction with long reads, followed by refinement with short reads—ensured base-level accuracy.

**Figure 1:**
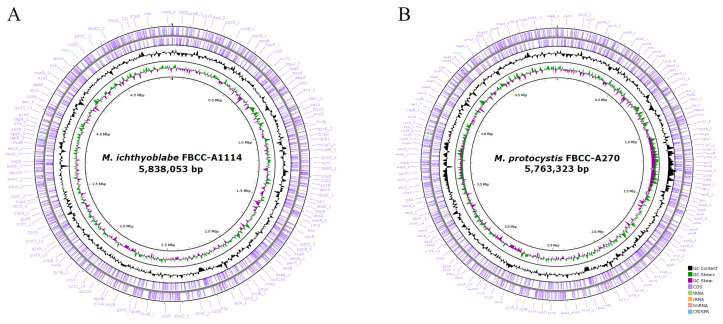
Circular genome maps of the two assembled strains. (**A**) *M. ichthyoblabe* FBCC-A1114 and (**B**) *M. protocystis* FBCC-A270. Each map displays genomic features including GC content and gene distribution. The strain name and total chromosome size are indicated at the center of each map. Individual tracks are color-coded according to feature type.

The final assemblies were characterized as follows: The genome of *M. ichthyoblabe* FBCC-A1114 was 5,838,053 bp in size with a GC content of 42.3 mol%. The assembly exhibited high genome completeness, with BUSCO and CheckM2 scores of 99.4% and 99.62%, respectively. Gene annotation identified 4706 coding sequences (CDSs), 4 rRNAs, 46 tRNAs, and 1 tmRNA. The genome of *M. protocystis* FBCC-A270 was 5763,323 bp in size (42.5 mol% GC content) with BUSCO and CheckM2 completeness scores of 99.3% and 99.92%. Annotation revealed 4701 CDSs, 4 rRNAs, 45 tRNAs, and 1 tmRNA. Both strains had a single circular chromosome, and no plasmids were detected. A summary of the genome features was provided in table (Table 1) and visualized as circular genome maps (Figure 1). In addition, the genome of *M. aeruginosa* FBCC-A68 was assembled and included in phylogenetic analysis. This strain consisted of a single circular chromosome of 5,864,213 bp with a GC content of 42.68 mol%. A total of 5553 CDSs and 51 RNAs (including 4 rRNAs, 46 tRNAs, and 1 tmRNA) were annotated, and no plasmids were detected.

In conclusion, the genomes of *M. ichthyoblabe* and *M. protocystis*, assembled for the first time in this study, exhibit high genome completeness and are suitable for further genomic studies. Species identification based on the tetra correlation search (TCS) using JSpeciesWS indicated high similarity of *M. ichthyoblabe* FBCC-A1114 to *M. aeruginosa* strains SPC777 (Z-score: 0.99948), TAIHU98 (0.99944), and PCC 7941 (0.99923). Similarly, *M. protocystis* FBCC-A270 showed close similarity to *M. aeruginosa* strains TAIHU98 (Z-score: 0.9995), SPC777 (0.99942), and PCC 7941 (0.99916). *M. aeruginosa* FBCC-A68 was found to have the highest similarity to *M. aeruginosa* NIES-843 (0.99801). These TCS values indicate that the newly assembled genomes belong to the genus *Microcystis*.

However, it should be noted that the JSpeciesWS reference database is currently biased toward *M. aeruginosa*, which limits an assessment of genome similarities across the diverse morphospecies. Therefore, the newly assembled complete genomes of *M. ichthyoblabe* and *M. protocystis* provide valuable genomic resources for advancing phylogenetic classification and functional studies in *Microcystis*.

### 3.2. Phylogenetic Analysis of Assembled Microcystis Strains

**Figure 2:**
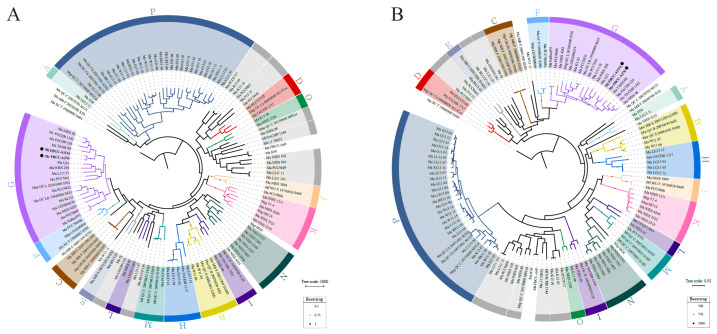
Rooted maximum likelihood phylogenetic trees of 125 *Microcystis* strains based on pan-genome and single-copy core genes. The tree was rooted using Ma_SC_T_19800800_S464, as identified in a prior comparative analysis of *Microcystis* and other cyanobacterial genomes. In addition, genospecies clusters supported by ANI and dDDH thresholds are color-coded; strains not assigned to any genospecies are shown in gray. (**A**) Tree based on gene presence/absence profiles from the pan-genome analysis. Bootstrap support values are shown as dot sizes, representing normalized values ranging from 0.5 to 1; only values ≥0.5 are displayed. (**B**) Tree constructed using alignments of 1448 single-copy core genes. Bootstrap values were calculated from 1000 replicates, and values ≥500 are indicated by dot sizes. The newly assembled strains, *M. ichthyoblabe* FBCC-A1114 and *M. protocystis* FBCC-A270, are grouped within genospecies G and are highlighted with a filled circle (●) adjacent to the strain name.

In this study, we applied a previously proposed genospecies classification framework to evaluate the molecular phylogeny of the assembled genomes. We employed the OGRI approach with genospecies boundaries defined by thresholds of ANI ≥ 0.970 and dDDH ≥ 0.750 for the same genospecies, and values should be lower than these thresholds for different genospecies [29]. Phylogenetic trees were constructed using gene presence/absence patterns (Figure 2A) and alignments of single-copy core genes (Figure 2B). The analysis included 122 available *Microcystis* genomes used in the previous study, along with three newly assembled genomes in this study (FBCC-A68, FBCC-A1114, and FBCC-A270). For clarity, the 125 strains in the figures are shown using abbreviated forms (Supplementary Table S2).

The pan-genome analysis identified a total of 21,230 genes, including 2070 core genes and 19,160 accessory genes. Among these, 1448 genes were identified as single-copy core genes conserved across all strains. Adding the three assembled strains resulted in minor changes in overall tree topology and strain positions, but the genospecies clustering pattern was maintained. Both *M. ichthyoblabe* FBCC-A1114 and *M. protocystis* FBCC-A270 were placed within genospecies G in both phylogenetic trees, and their placement was supported by strong bootstrap values (>900) in the single-copy core gene tree. OGRI analysis confirmed that these two strains shared ANI values ≥ 0.973 and dDDH ≥ 0.77 with other strains of genospecies G, while showing ANI values < 0.968 and dDDH < 0.737 with other genospecies, satisfying the threshold (Supplementary Table S3). In summary, molecular phylogenetic analysis supports the classification of both strains within genospecies G. On the other hand, *M. aeruginosa* FBCC-A68 did not belong to any genospecies.

Genospecies G includes diverse morphospecies such as *M. aeruginosa*, *M. ichthyoblabe*, *M. protocystis*, and *M. viridis*, which exhibit distinct features in terms of colony morphology, cell size, and mucilage characteristics. For example, *M. aeruginosa* typically forms irregularly shaped colonies composed of densely packed cells (~5 µm diameter) surrounded by well-defined mucilage. In contrast, *M. ichthyoblabe* exhibits sparsely distributed small cells with diffuse mucilage. *M. protocystis* forms spherical colonies consisting of cells ranging from 3.5–7.2 µm, while *M. viridis* displays cuboidal, packet-like colony structures [24,49].

However, the morphology of *Microcystis* is known to be highly plastic and responsive to environmental conditions, resulting in a wide spectrum of forms even within the same species. One of the factors contributing to this plasticity is extracellular polysaccharides (EPS). A previous study demonstrated that colony development varies according to EPS concentration in single *Microcystis* cells [50]. Control group formed ring-shaped colonies with a median diameter of 183 µm within 12 days, whereas high-EPS treated group formed irregularly shaped, loosely aggregated spherical colonies with a smaller median diameter of 130 µm. These observations suggest that EPS modulates intercellular adhesion and may constrain colony expansion, enabling environmentally induced morphological divergence.

In this study, despite morphological differentiation, phylogenetic analysis revealed that *M. ichthyoblabe* and *M. protocystis* belong to the same genospecies G, sharing high genome similarity, with ANI and dDDH values of 99% and 92%, respectively. This highlights the incongruence between morphological and molecular classifications within *Microcystis*. These findings demonstrate that conventional morphology-based classification is insufficient to reflect the species complexity and evolutionary relationships in *Microcystis*, thus emphasizing the need for a comprehensive review through a molecular-based phylogenetic framework.

### 3.3. Functional and Structural Complexity of Microcystis

**Figure 3:**
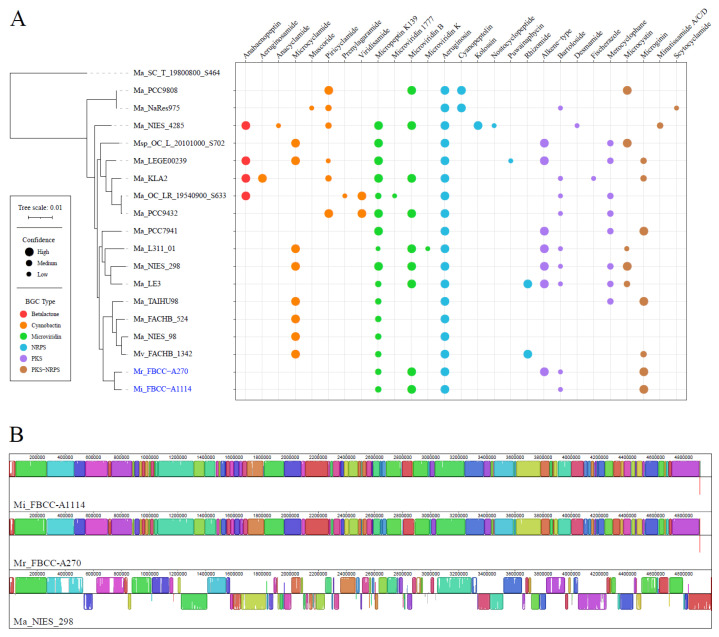
Comparison of BGC profiles and genome synteny among genospecies G strains. (**A**) Dot plot summarizing secondary metabolite biosynthetic gene clusters identified in genospecies G strains. The y-axis corresponds to the phylogeny based on single-copy core genes, and the x-axis indicates individual BGCs. Dot size reflects the confidence level of cluster prediction (low, medium, high), and dot color indicates BGC type (Betalactone, Cyanobactin, Microviridin, NRPS, PKS, and PKS–NRPS), as shown in the accompanying legend. *M. ichthyoblabe* FBCC-A1114 and *M. protocystis* FBCC-A270 are highlighted in blue. (**B**) Whole-genome synteny analysis among three genospecies G strains—FBCC-A1114, FBCC-A270, and *M. aeruginosa* NIES-298—based on whole-genome alignment. Locally collinear blocks (LCBs) represent regions of conserved gene order, with block similarity indicated within each LCB.

We analyzed the BGC composition and synteny among genospecies G strains to evaluate the functional and structural complexity within the same genospecies. AntiSMASH analysis revealed that most strains of genospecies G share BGCs for aeruginosin, cyanobactin, and microviridin (Figure 3A). These BGCs are known to have the following roles: Aeruginosins are serine protease inhibitors that disrupt digestive enzyme activity in predators [51,52], and cyanobactins are associated with oxidative stress resistance and antimicrobial activity [53]. In addition, microviridins serve as protease inhibitors that support survival during interbacterial competition [54]. The conservation of these BGCs suggests that genospecies G strains share common adaptive strategies for predator defense and stress tolerance. In contrast, *M. ichthyoblabe* FBCC-A1114 and *M. protocystis* FBCC-A270 lack both microcystin and cyanobactin BGCs. Instead, both strains possess the microginin BGC, which is known to broadly inhibit metalloproteases and certain serine proteases [55,56]. The presence of microginin may functionally compensate for the absence of cyanobactin and microcystin, potentially serving as an alternative defense strategy [57]. These BGC diversity for survival strategies illustrate the adaptability of *Microcystis* to different environments.

In addition to functional analysis, we analyzed genome structure within genospecies G by performing synteny analysis with *M. aeruginosa* NIES-298, the only strain in the group with a complete genome (Figure 3B). *M. ichthyoblabe* FBCC-A1114 and *M. protocystis* FBCC-A270 exhibited high conservation of gene order, whereas *M. aeruginosa* NIES-298 showed significant inversions and translocations. This result demonstrates that plasticity of genome structure can actively occur even within the same genospecies. In particular, genomic rearrangements such as inversions and translocations can modify gene order, thereby altering gene expression profiles, gene dosage, or even the generating novel gene fusions, which are known mechanisms for rapid adaptation in bacteria [58,59]. These structural changes can provide a selective advantage in specific environmental niches, for instance, by optimizing metabolic pathways for nutrient acquisition, enhancing stress response mechanisms, or facilitating evasion from predators, thereby contributing to ecological differentiation [60,61]. However, the assessment of structural complexity within genospecies G genomes remains limited due to the lack of complete genome for most strains. Expanding the dataset with complete genomes will allow us to understand patterns of structural differences and their ecological implications.

Differences in BGC profiles among strains within the same genospecies reflect the diversification of adaptive strategies such as stress tolerance and competitive survival mechanisms. Notably, the two newly sequenced strains, *M. ichthyoblabe* FBCC-A1114 and *M. protocystis* FBCC-A270, were isolated from geographically proximate locations. Despite being classified as distinct morphospecies, they exhibit high genomic similarity and largely conserved synteny. This indicates that, while their fundamental genomic architecture is conserved, minor differences in gene content may facilitate adaptation to closely related yet distinct micro-niches, or environmentally induced phenotypic divergence within a shared ecological context. These findings support the genomic complexity and ecological divergence of *Microcystis*. Further molecular-based studies will help elucidate the evolutionary trajectories of these adaptations, advancing our understanding of *Microcystis*.

## 4. Conclusions

Cyanobacteria of the genus *Microcystis* are major contributors to HABs, posing significant ecological and public health risks. However, morphology-based classification remains a challenging due to the morphological plasticity of *Microcystis* under diverse environmental conditions. In addition, the lack of genome data for morphospecies other than *M. aeruginosa* has hindered a comprehensive understanding of the species complexity and evolutionary dynamics. To address this gap, we assembled the first complete genome sequences of *M. ichthyoblabe* and *M. protocystis*, aiming to advance our understanding of the complexity in *Microcystis* through comparative genome analysis.

The assembled genomes of *M. ichthyoblabe* FBCC-A1114 and *M. protocystis* FBCC-A270 are approximately 5.8 Mbp and 5.7 Mbp in size, respectively, and exhibit high completeness (>99%). Comprehensive classification analysis based on pan-genome and OGRI thresholds identified that both strains belong to genospecies G, along with *M. aeruginosa* and *M. viridis*. Interestingly, the two strains displayed high genome similarity with ANI values of 99% and dDDH values of 92%, indicating the closest evolutionary relationship, despite their morphological differences.

Analysis of BGCs showed that most genospecies G strains share conserved gene clusters for aeruginosin, cyanobactin, and microviridin biosynthesis, supporting a common adaptive strategy for defense against predators and environmental stress. However, *M. ichthyoblabe* FBCC-A1114 and *M. protocystis* FBCC-A270 lacked microcystin and cyanobactin clusters, indicating a nontoxic ecotype profile. Instead, the presence of the microginin cluster in these strains suggests an alternative survival strategy, contributing to functional complexity within genospecies G.

Synteny analysis revealed a high level of gene order conservation between the two strains, while extensive genome rearrangements were observed in *M. aeruginosa* NIES-298, indicating plasticity of genome structure even within the same genospecies. These findings highlight an incongruence between morphological classification and actual genome-based features, emphasizing the need for a comprehensive approach in studying highly plastic *Microcystis*.

Despite these insights, most *Microcystis* strains still lack complete genome sequences, limiting assessments of functional and structural complexity. In addition, the presence of BGCs is based on in silico predictions. Therefore, further research involving metabolomics, RNA-seq, and comparative transcriptomics under diverse environmental conditions is essential to functionally validate gene cluster expression and elucidate mechanisms of environmental adaptation. The continued expansion of complete genome sequences from diverse morphospecies, alongside studies linking genomic traits to ecological factors, will enable a deeper understanding of ecological divergence and evolutionary dynamics within the genus *Microcystis*. This study provides a foundational genomic resource for the genus *Microcystis* and supports future studies on its complexity and ecological adaptability.

## Figures and Tables

**Table 1:** Genome contents of *M. ichthyoblabe* FBCC-A1114 and *M. protocystis* FBCC-A270. Summary of genome size, GC content, sequence structure, and gene annotation features of the two strains.

Strain	FBCC-A1114	FBCC-A270
Species Name	*Microcystis ichthyoblabe*	*Microcystis protocystis*
NCBI Taxonomy ID	1125	
Domain	Bacteria	
Taxonomy	Bacteria; Bacillati; Cyanobacteriota/Melainabacteria group; Cyanobacteriota; Cyanophyceae; Oscillatoriophycideae; Chroococcales; Microcystaceae; Microcystis	
Genome Size (bp)	5,838,053	5,763,323
GC content (mol%)	42.3	42.5
Number of Genome Sequences	1 Circular (Single chromosomal DNA without plasmid)	
Number of Plasmids	0	
Number of Coding Sequences	4706	4701
Number of rRNAs	4	4
Number of tRNAs (tmRNA)	46 (1)	45 (1)

## Data Availability

The genome sequences generated in this study have been deposited in NCBI under BioProject accession number PRJNA1262101. All code used in the analysis is available at https://github.com/Jin-cat/Microcystis_analysis, accessed on 15 June 2025.

## Supplementary Materials

The following supporting information can be downloaded at: https://www.mdpi.com/article/10.3390/microorganisms13071693/s1. Table S1: The number of raw bases before and after adapter trimming for *M. aeruginosa* FBCC-A68, *M. protocystis* FBCC-A270, and *M. ichthyoblabe* FBCC-A1114. Table S2: Information on *Microcystis* Strains Used for Analysis and Their Abbreviations. Table S3: Pairwise ANI and dDDH Values of *Microcystis* Genomes Used for Genospecies Assignment.

## Abbreviations

The following abbreviations are used in this manuscript:

HAB	Harmful Algal Bloom
ITS	Internal Transcribed Spacer
OGRI	Orthologous average Genome Relatedness Index
ANI	Average Nucleotide Identity
dDDH	digital DNA-DNA Hybridization
BGC	Biosynthesis Gene Cluster
CDS	Coding Sequence
TCS	Tetra Correlation Search
EPS	Extracellular Polysaccharide
PEPPAN	Phylogenetic Estimation of Pangenome using PEPPAN
BUSCO	Benchmarking Universal Single-Copy Orthologs
